# Gender-Specific Population Attributable Fractions for Cardiovascular Disease and All-Cause Mortality Associated with Living Arrangement in Community-Dwelling Older People

**DOI:** 10.1007/s11606-025-09648-7

**Published:** 2025-06-25

**Authors:** Achamyeleh Birhanu Teshale, Htet Lin Htun, Alice J. Owen, JR Baker, Irja Isaksen, Rosanne Freak-Poli

**Affiliations:** 1 School of Public Health and Preventive Medicine, Monash University, Melbourne, VIC Australia; 2 Department of Epidemiology and Biostatistics, Institute of Public Health, College of Medicine and Health Sciences, University of Gondar, Gondar, Ethiopia; 3 Faculty of Health, Southern Cross University, Bilinga, QLD Australia; 4 Primary & Community Care Services Limited, Thornleigh, NSW Australia; 5 Australian Social Prescribing Institute of Research and Education, Surry Hills, NSW Australia; 6 Department of Physiology, Monash University, Melbourne, VIC Australia; 7 School of Clinical Sciences at Monash Health, Monash University, Melbourne, VIC Australia

**Keywords:** cardiovascular disease, disease burden, all-cause mortality, living arrangement, social connection

## Abstract

**Background:**

Living alone poses significant cardiovascular disease (CVD) and mortality risks. The population attributable fraction (PAF) quantifies the proportion of disease burden attributable to a specific risk factor. This study aims to estimate the PAF for CVD and all-cause mortality related to living completely alone. Additionally, the study examined the PAFs associated with not living with a partner/spouse.

**Methods:**

This study used longitudinal data from the Aspirin in Reducing Events in the Elderly (ASPREE) study and its sub-study, the ASPREE Longitudinal Study of Older Persons (ALSOP), which included 5853 men and 6998 women. The participants were community-dwelling healthy adults aged 70 + years without CVD, dementia, or significant physical disabilities. Adjusting for social determinants and traditional risk factors, the gender-specific PAFs of CVD and all-cause mortality attributable to living completely alone and not living with a partner/spouse were determined.

**Results:**

Among women, 13.5% (95% CI: 6.3%, 22.4%) of CVD events could be attributed to living completely alone, and 14.1% (95% CI: 4.6%, 25.2%) to not living with a partner/spouse. For all-cause mortality in women, the corresponding PAFs were 9.8% (95% CI: 3.7%, 16.6%) and 12.3% (95% CI: 5.8%, 20.8%), respectively. In contrast, among men, only the PAF between not living with a partner/spouse and all-cause mortality reached statistical significance (PAF = 6.0%; 95% CI: 1.7%, 10.2%). The remaining PAF estimates for CVD events and all-cause mortality were not statistically significant.

**Conclusion:**

The observed gender differences in CVD and all-cause mortality related to living alone highlight the need for tailored public health interventions to meet gender-specific needs for social connectedness.

**Supplementary Information:**

The online version contains supplementary material available at 10.1007/s11606-025-09648-7.

## BACKGROUND

Living alone is becoming increasingly common in developed countries. At least one in five people live alone but can be as high as half of the population. For example, regardless of age, Scandinavia reports the highest rates of living alone, with figures ranging from 39% in Denmark to 49% in Sweden. In Western European countries, the rates vary from 34% in Belgium to 40% in Germany. In Southern Europe, the percentages are lower, ranging from 23% in Spain to 28% in Italy. Living alone is also common in the United States (29%), Australia (24%), and New Zealand (22%).^1^ The trend is expected to continue as societies develop and age.^1^

Living alone is recognised as a significant social and health risk factor.^2-4^ A systematic review and meta-analysis indicated that adults living alone have a 48% increased risk of all-cause mortality compared to those living with others.^4^ Another meta-analysis of community-dwelling adult cohorts demonstrated that living alone is associated with a 22% increased risk of CVD and a 23% higher risk of cardiovascular mortality.^3^ Similarly, another systematic review found that living alone is associated with a 15% increased risk of all-cause mortality.^2^ A three-decade longitudinal study also identified living alone as an independent risk factor for both all-cause and cardiovascular mortality.^5^ However, most of these studies only included younger or middle-aged adults, with limited consideration of older populations.^6-13^ Regarding gender differences, two meta-analytic studies and a number of individual studies have identified living alone to be associated with higher health risks for men.^3,10,11,14-16^ However, others report no gender-specific associations,^13,17^ similar associations across genders,^16^ or stronger associations with adverse health outcomes among women.^18,19^ The variation in the impact of living alone across studies and genders may stem from disparities in cultural norms, differences in population characteristics, methodological approaches, and the residual confounding factors.

Despite the evidence for living alone as a public health concern,^4^ and our prior study on healthy older adults identifying living alone as a key predictor of CVD risk,^19^ the population attributable fractions (PAF) for CVD and all-cause mortality remain underexplored. The PAF is a metric that quantifies the proportion of disease burden, such as CVD and overall mortality, attributable to a specific risk factor, such as living alone, within a population.^20^ Essentially, the PAF indicates the percentage of diseases or deaths that could potentially be prevented by eliminating the risk factor or reducing its prevalence. This population-level perspective is critical for informing public health policy and intervention strategies.^20-23^ This present study also incorporated gender-disaggregated analysis, building on the above studies that highlighted gender differences. This is further motivated by the fact that CVD and mortality impact men and women differently, exhibiting significant disparities in prevalence, risk factors, symptoms, and prognosis.^24,25^ Thus, this study aimed to assess the gender-specific PAFs for CVD and all-cause mortality associated with living arrangements in community-dwelling older adults. To explore living arrangements, we first assessed living completely alone, and secondly, not living with a partner/spouse.

## METHODS

### Data Source and Study Population

This study utilised the Aspirin in Reducing Events in the Elderly (ASPREE)^26^; the ASPREE-eXTension (ASPREE-XT),^27^ which is the post-trial observational study of the ASPREE; and the ASPREE Longitudinal Study of Older Persons (ALSOP).^28^ ASPREE was a randomised controlled trial to assess the effect of low-dose aspirin among 19,114 older adults aged 70 + from Australia and the United States (US), including US minorities aged 65 +. ASPREE recruited healthy individuals who were free of major physical disability, CVD events, and dementia. Recruitment was undertaken from 2010 to 2014. After a median follow-up period of 4.7 years (ending on 12 June 2017), the ASPREE trial ended due to findings that aspirin had no impact on health outcomes, including CVD and CVD mortality.^29,30^ Subsequently, notification letters were sent to all participants to inform them of the trial’s conclusion. Between June 12, 2017, and January 31, 2018, all participants originally enrolled in ASPREE who had not withdrawn or passed away were invited to continue with an annual observational follow-up in ASPREE-XT. ALSOP is a sub-study of the ASPREE trial, and thus far, it comprises approximately 90% of the Australian participants from the larger ASPREE cohort. This study was limited to ALSOP cohort participants due to the availability of living arrangement data in the ALSOP data collection.

### Variables of the Study

#### Outcomes

CVD and all-cause mortality were the outcome variables. CVD was measured as a composite of fatal coronary heart disease, stroke (fatal or non-fatal), myocardial infarction (non-fatal), and hospitalisation due to heart failure.^32^ All-cause mortality includes cancer-related deaths, death due to clinically significant bleeding, cardiovascular (including stroke) death, and other deaths.^29^ Both CVD and all-cause mortality were adjudicated by panels of clinical experts.^29,32^ All participants were monitored until they experienced CVD events, death, loss to follow-up, or completed their fourth annual ASPREE-XT visit (between 2021 and 2022), whichever came first.^22^

#### Exposure Variable

Living arrangement is the main exposure of interest. At ALSOP enrolment, participants indicated whether they live alone or with others (including partner/spouse, children, grandchildren, other relatives, friends, or others) to the question, “Who do you currently live with?” Two types of exposure were then formed, and the variable was renamed as:

1. *Living completely alone*, categorised as “no” for those who live with anyone else and “yes” for those who live completely alone.^31^
2. *Living with a partner/spouse* as a secondary exposure variable, marked “yes” if participants lived with a partner or spouse and “no” otherwise (living completely alone or with someone other than a partner/spouse). This distinction allows for a more specific analysis than the general *living completely alone* variable, focusing on how partnership influences CVD and all-cause mortality. For consistency, estimates for *not living with a partner/spouse* were provided to align conceptually with the *living completely alone* variable.

Although these two variables are correlated, we treated them separately for two primary reasons: first, our previous study revealed different findings for men and women, indicating that living with a partner/spouse was associated with a reduced risk of CVD among men, while both living with someone else and living with a partner/spouse reduced CVD risk among women.^19^ Second, living with a partner or spouse is generally regarded as the most intimate cohabitation arrangement by combining emotional, physical, and practical closeness in ways that other living arrangements do not. This could in turn significantly improve both physical and mental health.

#### Covariates

Both social determinants of health (SDoH) and traditional factors were considered as covariates.

SDoH variables were grouped based on the Healthy People 2030 framework^33^ and outlined as follows:

1. *Economic stability*: Includes employment, household income, and homeownership.
2. *Education access and quality*: Encompassing education status and first language.
3. *Social and community context*: Includes social network, social participation, volunteering, optimism, stressful life events, hobby engagement, and depression
4. *Health care access and quality*: Considered health insurance
5. *The neighbourhood and built environment*: Includes remoteness, socioeconomic indexes for areas (SEIFA), and satisfaction with transportation.

The traditional covariates in this study were age, smoking, hypertension, diabetes, dyslipidaemia, and renal function measures (urine albumin-to-creatinine ratio (ACR) and estimated glomerular filtration rate (eGFR)). The details about the measurement and categorisation of the variables are elaborated in Supplementary table S1 and our published article.^34^ Building on prior research from the ASPREE cohort, which revealed an increased risk of all-cause mortality among individuals taking aspirin compared to placebo,^29^ treatment assignment (aspirin vs. placebo) was also included as a covariate in the analysis of the relationship between exposure variables and all-cause mortality. However, because aspirin showed no effect on CVD in this cohort,^29,30^ it was excluded as a covariate when assessing the relationship between the exposure variables and CVD risk.

### Statistical Analysis

Stata v.17 and R v.4.3.3 were used for statistical analyses. All the analyses were gender-disaggregated (i.e. men vs. women, as no other gender identities were recorded). Continuous variables were reported using mean and standard deviation (SD) or median and interquartile range (IQR), as appropriate. Categorical variables were reported using frequency and percentage. Missing values were imputed using the *missForest* package,^35^ which employs the random forest algorithm for imputation.

The Cox proportional hazards model was used to assess the association between our exposure variables and CVD and all-cause mortality. The proportional hazards assumption was checked using the Schoenfeld residual test.^36^ Then, the PAFs of CVD and all-cause mortality due to living alone and not living with a partner/spouse were assessed. To estimate the PAFs, the *graphPAF* package in R was used.^37^ The *graphPAF* package is intended to estimate and display different types of PAF and impact fractions, which measure the disease burden attributable to a certain risk factor(s). Subsequently, we reported the proportion of CVD events and all-cause mortality that could have potentially been prevented if no individuals within the cohort were living alone, including if someone was living with a partner/spouse. At baseline (time = 0), no participants experienced CVD events or all-cause mortality. Over time, the cumulative incidence and proportion of these outcomes that could potentially be prevented will evolve. Therefore, in this study, different time points were specified to consider PAF(t). A statistically significant association was determined when the confidence interval (CI) for the hazard ratios (HRs) and the PAFs did not include 1 and 0, respectively.

## RESULTS

### Participant Characteristics

**Figure 1:**
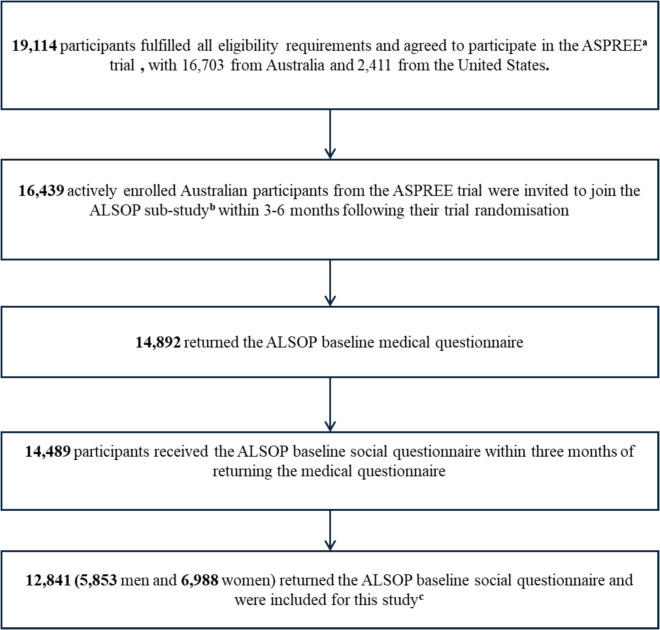
Flowchart of participant selection. The superscript letter a denotes that following a median of 4.7 years of follow-up, the ASPREE trial has transitioned into an observational follow-up study known as the ASPREE-eXTension (ASPREE-XT). The superscript letter b denotes that the ALSOP sub-study comprises both the ALSOP medical questionnaire and social questionnaire. Eligibility for the social questionnaire was contingent upon participants first completing and returning the medical questionnaire; individuals who did not return the medical questionnaire were ineligible to participate in the social questionnaire. The superscript letter c denotes that 1) the *missForest* imputation method was utilised for imputing missing observations for both men and women separately, and 2) to mitigate the issue of temporality, since living arrangement was collected in the ALSOP sub-study, CVD cases that occurred before the distribution of the ALSOP questionnaire (*n* = 31 for men and *n* = 24 for women) were excluded.

This study included 12,841 Australians, 5853 men and 6988 women aged 70 + years, as illustrated in Fig. 1. The median age of men and women participants was 73.9 years [IQR, 71.6–77.4] and 74.1 years [IQR, 71.8–77.8], respectively. The characteristics of the study participants based on traditional and SDoH variables are illustrated in Table 1.

**Table 1:** Baseline Characteristics of Participants

Characteristics	Men (*n* = 5853)	Women (*n* = 6988)
**Demographic and clinical risk factors**^**a**^		
Age in years (median [IQR])	73.9 [71.6–77.4]	74.1 [71.8–77.8]
Treatment assignment (%)		
Aspirin	2949 (50.1)	3540 (50.5)
Placebo	2935 (49.9)	3865 (49.5)
Hypertension (%)		
No	1435 (24.5)	1861 (26.6)
Yes	4418 (75.5)	5127 (73.4)
Diabetes		
No	5168 (88.3)	6443 (92.2)
Yes	685 (11.7)	545 (7.8)
Dyslipidaemia (%)		
No	2571 (43.9)	1613 (23.1)
Yes	3282 (56.1)	5375 (76.9)
Current smoking (%)		
No	5657 (96.6)	6828 (97.7)
Yes	196 (3.4)	160 (2.3)
Urine ACR in mg/mmol (median [IQR]))	0.7 [0.4–1.3]	0.9 [0.5–1.5]
Estimated GFR in ml/min/1.73m^2^ (median [IQR]))	74.2 [64.3–83.5]	73.9 [63.6–84.0]
**Social determinants of health**^**a**^		
a) Economic stability		
Employment (%)		
No	5108 (87.3)	6594 (94.4)
Yes	745 (12.7)	394 (5.6)
Income (%)		
Low	3821 (65.3)	5016 (71.8)
High	2032 (34.7)	1972 (28.2)
Home ownership (%)		
No	464 (7.9)	715 (10.2)
Yes	5389 (92.1)	6273 (89.8)
b) Education quality and access		
Education status (%)		
Low (≤ 12 years)	3249 (55.5)	4337 (62.1)
High (> 12 years)	2604 (44.5)	2651 (37.9)
English as first language (%)		
No	239 (4.1)	193 (2.8)
Yes	5614 (95.9)	6795 (97.2)
c) Social and community context		
Social network (%)		
Low	666 (11.4)	864 (12.4)
High	5187 (88.6)	6124 (87.6)
Social participation (%)		
Low	1084 (18.5)	766 (11.0)
High	4769 (81.5)	6222 (89.0)
Volunteering (%)		
No	1457 (24.9)	702 (10.1)
Yes	4396 (75.1)	6286 (89.9)
Expectations and attitudes (optimism) (%)		
Less optimistic	1212 (20.7)	1281 (18.3)
Highly optimistic	4641 (79.3)	5707 (81.7)
Experiencing stressful life events (%)		
None	2038 (34.8)	2108 (30.2)
1	2118 (36.2)	2587 (37.0)
2	1096 (18.7)	1449 (20.7)
3 or more	601 (10.3)	844 (12.1)
Hobby engagement (%)		
Less engaged	962 (16.4)	298 (4.3)
Highly engaged	4891 (83.6)	6690 (95.7)
Depression (%)		
No	5422 (92.6)	6282 (89.9)
Yes	431 (7.4)	706 (10.1)
d) Health care access and quality		
Health insurance (%)		
Not insured	270 (4.6)	331 (4.7)
Insured	5583 (95.4)	6657 (95.3)
e) Neighbourhood and built environment		
Remoteness (%)		
Major cities	2085 (35.6)	2469 (35.3)
Inner regions	3107 (53.1)	3719 (53.2)
Outer regions	661 (11.3)	800 (11.5)
Socioeconomic indexes for areas (SEIFA) (%)		
1 st quintile (most deprived)	910 (15.5)	1134 (16.2)
2nd quintile	999 (17.1)	1175 (16.8)
3rd quintile	1067 (18.2)	1329 (19.0)
4th quintile	1110 (19.0)	1362 (19.5)
5th quintile (most affluent)	1767 (30.2)	1988 (28.5)
Satisfaction with transportation (%)		
No	183 (3.1)	335 (4.8)
Yes	5670 (96.9)	6653 (95.2)

^a^For a more detailed understanding of how the variables were categorised, please refer to Supplementary Table S1

### Incidence of CVD and All-Cause Mortality

Participants were followed for a maximum duration of 12 years, with the median follow-up period of approximately 8.3 years for both men and women. Among men, the incidence rate of CVD and all-cause mortality was 14.5 (95% CI: 13.4, 15.6) and 20.7 (95% CI: 19.5, 22.0) events per 1000 person-years, respectively (Table 2). The total analysis time at risk was 46,618 for CVD events and 49,742 years for all-cause mortality. For women, the rate of CVD was 9.5 (95% CI: 8.7, 10.3) and all-cause mortality was 13.5 (95% CI: 12.6, 14.5) events per 1000 person-years (Table 2). The total analysis time at risk was 57,749 for CVD events and 61,287 years for all-cause mortality.

**Table 2:** Living Completely Alone and Not Living with a Partner/Spouse and Incidence of CVD and All-Cause Mortality

Exposure	Frequency (%)	Cardiovascular disease			All-cause mortality		
		**No, *n* (%)**	**Yes**		**No, *n* (%)**	**Yes**	
			***n* (%)**	**IR (95% CI)**		***n* (%)**	**IR (95% CI)**
Men, *n* (%)	5853 (100)	5177 (88.5)	676 (12.0)	14.5 (13.4, 15.6)	4822 (82.4)	1031 (17.6)	20.7 (19.5, 22.0)
Living completely alone							
No, *n* (%)	4880 (83.4)	4347 (89.1)	533 (10.9)	13.6 (12.5, 14.8)	4106 (84.1)	774 (15.9)	18.5 (17.3, 19.9)
Yes, *n* (%)	973 (16.6)	830 (85.3)	143 (14.7)	19.5 (15.5, 23.0)	716 (73.6)	257 (26.4)	32.3 (28.6, 36.6)
Living with a spouse/partner							
No, *n* (%)	1215 (20.8)	1039 (85.5)	176 (14.5)	19.2 (16.6, 22.3)	901 (74.2)	314 (25.8)	31.7 (28.4, 35.4)
Yes, *n* (%)	4638 (79.2)	4138 (89.2)	500 (10.8)	13.4 (12.2, 14.6)	3921 (84.5)	717 (15.5)	18.0 (16.7, 19.4)
Women, *n* (%)	6988 (100)	6439 (92.1)	549 (7.9)	9.5 (8.7, 10.3)	6159 (88.1)	829 (11.9)	13.5 (12.6, 14.5)
Living completely alone							
No, *n* (%)	4132 (59.1)	3880 (93.9)	252 (6.1)	7.3 (6.4, 8.3)	3757 (90.9)	375 (9.1)	10.3 (9.3, 11.4)
Yes, *n* (%)	2856 (40.9)	2559 (89.6)	297 (10.4)	12.8 (11.4, 14.3)	2402 (84.1)	454 (15.9)	18.2 (16.6, 20.0)
Living with a spouse/partner							
No, *n* (%)	3389 (48.5)	3050 (90.0)	339 (10.0)	12.3 (11.1, 13.7)	2860 (84.4)	529 (15.6)	17.9 (16.4, 19.5)
Yes, *n* (%)	3599 (51.5)	3389 (94.2)	210 (5.8)	7.0 (6.1, 8.0)	3299 (91.7)	300 (8.3)	9.5 (8.4, 10.6)

**Abbreviations:** *CI*, confidence interval; *IR*, incident rate

### Living Completely Alone and the Risk of CVD and All-Cause Mortality

In this study, 16.6% of men and 40.9% of women were living completely alone. Men and women who lived completely alone had a higher incidence of CVD and all-cause mortality (Table 2). In the Cox model, for men, living completely alone was not associated with CVD or all-cause mortality. However, for women, it was significantly associated with higher risks of both CVD (adjusted HR (aHR): 1.32; 95% CI: 1.11, 1.58) and all-cause mortality (aHR: 1.21; 95% CI: 1.05, 1.40) (Table 3).

**Table 3:** The Cox Proportional Hazards Model in Assessing the Association Between Living Completely Alone, as well as Not Living with a Partner/Spouse, and CVD and All-Cause Mortality

Outcomes	Exposures	aHR (95% CI)^a^
**Men**		
Cardiovascular disease	Living completely alone	
	No	Reference
	Yes	1.11 (0.93, 1.38)
	Not living with a partner/spouse	
	No	Reference
	Yes	1.17 (0.97, 1.41)
All-cause mortality^b^	Living completely alone	
	No	Reference
	Yes	1.15 (0.99, 1.34)
	Not living with a partner/spouse	
	No	Reference
	Yes	**1.22 (1.05, 1.40)**
**Women**		
Cardiovascular disease	Living completely alone	
	No	Reference
	Yes	**1.32 (1.11, 1.58)**
	Not living with a partner/spouse	
	No	Reference
	Yes	**1.29 (1.07, 1.55)**
All-cause mortality^b^	Living completely alone	
	No	Reference
	Yes	**1.21 (1.05, 1.40)**
	Not living with a partner/spouse	
	No	Reference
	Yes	**1.23 (1.06, 1.44)**

^a^The hazard ratios were adjusted for both social determinants of health (variables from economic stability, education, social and community context, neighbourhood and built environment, and health care quality and access themes) and traditional CVD risk factors (age, smoking, hypertension, diabetes, dyslipidaemia, urine albumin-to-creatinine ratio, and estimated glomerular filtration rate). Statistically significant findings are highlighted in bold

^b^The hazard ratios for all-cause mortality were also adjusted for treatment assignment (Aspirin vs. placebo)

**Abbreviations:** *CI*, confidence interval; *aHR*, adjusted hazard ratios

### Not Living with a Partner/Spouse and Risk of CVD and All-Cause Mortality

20.8% of men and 48.5% of women were not living with a partner/spouse. Men and women who were not living with a spouse or partner had a higher incidence of CVD and all-cause mortality (Table 2). In the Cox model, not living with a partner/spouse was associated with a 22% and 23% higher risk of all-cause mortality for men (aHR: 1.22; 95% CI: 1.05, 1.40) and women (aHR: 1.23; 95% CI: 1.06, 1.44), respectively. Not living with a partner/spouse was associated with the risk of CVD among women only (aHR: 1.29; 95% CI: 1.07, 1.55) (Table 3).

### PAF for CVD and All-Cause Mortality Associated with Living Completely Alone

Among men, the PAF for CVD attributable to living completely alone was not statistically significant (aPAF = 2.9%; 95% CI: − 1.2%, 7.1%, at all timepoints). In contrast, for women, this PAF was statistically significant at all timepoints (aPAF = 13.5%; 95% CI: 6.3%, 22.4%) (Table 4). Regarding all-cause mortality, among women, the proportion of all-cause mortality attributable to living completely alone was statistically significant (aPAF = 9.8%; 95% CI: 1.9%, 17.6%, at all timepoints). While among men, this PAF was not statistically significant (aPAF = 3.7%; 95% CI: − 0.8%, 7.6%, at all timepoints).

**Table 4:** Population Attributable Fraction for CVD and All-Cause Mortality Attributable to Living Completely Alone and Not Living with a Partner/Spouse

Outcomes	aPAF (95% CI)^a^				
	**Year 1**	**Year 3**	**Year 5**	**Year 10**	**Year ~ 12**
**Men**					
Cardiovascular disease	Living completely alone				
	2.9 (− 1.2, 7.1)	2.9 (− 1.2, 7.0)	2.9 (− 1.2, 7.0)	2.9 (− 1.2, 7.0)	2.9 (− 1.2, 7.0)
	Not living with a partner/spouse				
	4.3 (− 0.1, 8.8)	4.3 (− 0.1, 8.8)	4.3 (− 0.1, 8.8)	4.3 (− 0.1, 8.8)	4.3 (− 0.1, 8.8)
All-cause mortality^b^	Living completely alone				
	**3.7 (− 0.8, 7.6)**	**3.7 (− 0.8, 7.6)**	**3.7 (− 0.8, 7.6)**	**3.7 (− 0.8, 7.6)**	**3.7 (− 0.8, 7.6)**
	Not living with a partner/spouse				
	**6.0 (1.7, 10.2)**	**6.0 (1.7, 10.2)**	**6.0 (1.7, 10.2)**	**6.0 (1.7, 10.2)**	**6.0 (1.7, 10.2)**
**Women**					
Cardiovascular disease	Living completely alone				
	**13.5 (6.2, 22.3)**	**13.5 (6.3, 22.4)**	**13.5 (6.3, 22.4)**	**13.5 (6.3, 22.4)**	**13.5 (6.3, 22.4)**
	Not living with a partner/spouse				
	**14.1 (4.6, 24.9)**	**14.1 (4.6, 25.2)**	**14.1 (4.6, 25.2)**	**14.1 (4.6, 25.2)**	**14.1 (4.6, 25.2)**
All-cause mortality^b^	Living completely alone				
	**9.8 (3.7, 16.6)**	**9.8 (3.7, 16.6)**	**9.8 (3.7, 16.6)**	**9.8 (3.7, 16.6)**	**9.8 (3.7, 16.6)**
	Not living with a partner/spouse				
	**12.3 (5.8, 20.8)**	**12.3 (5.8, 20.8)**	**12.3 (5.8, 20.8)**	**12.3 (5.8, 20.8)**	**12.3 (5.8, 20.8)**

^a^The population attribution fractions were adjusted for both social determinants of health (variables from economic stability, education, social and community context, neighbourhood and built environment, and health care quality and access themes) and traditional CVD risk factors (age, smoking, hypertension, diabetes, dyslipidaemia, urine albumin-to-creatinine ratio, and estimated glomerular filtration rate). The PAFs and the CIs are expressed in percentages. Statistically significant findings are highlighted in bold

^b^The population attributable fractions for all-cause mortality are also adjusted for treatment assignment (Aspirin vs. placebo)

**Abbreviations:** *CI*, confidence interval; *aPAF*, adjusted population attributable fraction

### PAF for CVD and All-Cause Mortality Associated with Not Living with a Partner/Spouse

Similarly, the PAF for CVD associated with not living with a partner/spouse was statistically significant among women (aPAF = 14.1%; 95% CI: 4.6%, ~ 25.0%, at all timepoints) and was not statistically significant among men (aPAF = 4.3%; 95% CI: − 0.1%, 8.8%, at all timepoints). The PAF for all-cause mortality associated with not living with a partner/spouse was statistically significant among women (aPAF = 12.3%; 95% CI: 5.8%, 20.8%) and men (aPAF = 6.0%; 95% CI: 1.7%, 10.2%) (Table 4).

## DISCUSSION

### Main Findings

This study demonstrates variable PAFs for CVD and all-cause mortality associated with living completely alone and not living with a partner/spouse among older Australian men and women. In this cohort of older adults, the proportions of those living completely alone and not living with a partner/spouse were more than two times higher among women compared to men, which was not unexpected given the longer life expectancy for women compared to men in Australia.^38^ In brief, it is expected that women have a higher prevalence, as they generally live longer than men.^39-41^ While living completely alone was associated with increased risk of CVD and all-cause mortality, it only reached statistical significance for women, but not for men. The PAFs for CVD and all-cause mortality associated with living completely alone and not living with a partner/spouse were higher and statistically significant for women. Among men, only the PAF for all-cause mortality associated with not living with a partner/spouse was found to be statistically significant.

In the subsequent sections, we will place our findings in context by examining how they align with existing literature, exploring their implications for public health and policy, and outlining potential future directions.

### PAFs for CVD and All-Cause Mortality Attributable to Living Completely Alone

As no study has previously collated the PAFs for CVD and all-cause mortality attributable to living alone, we have calculated the PAFs from studies that explored gender-specific associations between living alone and CVD and all-cause mortality. The PAFs were estimated using Levin’s formula,^42^ based on the reported relative risk, odds ratio, or hazard ratio from previous studies (**Table S2)**. This approach was applied to provide a comparison of our PAF findings with the existing literature.^8,10,14-16,18,43-48^ Many of these studies revealed a statistically significant association between living alone and CVD and all-cause mortality among men, which then created higher PAFs among men compared to women. However, these studies looked at men and women of all ages. Aligned with our findings, a study conducted in Denmark that also focused on adults aged 70 + years reported that the all-cause mortality attributable to living alone was significantly higher among women compared to men.^18^

Various factors can modify the impact of social determinants, such as living alone, on CVD among women. One key factor is financial strain since women on average earn less than men and may face greater financial challenges when living alone, which can compound over a lifetime and then have greater influences on health in later life.^49-51^ Another factor is cultural expectations. In certain societies, there are stronger cultural expectations for women to be in partnerships and therefore, living alone may create more social stress for women compared to men.

### PAFs for CVD and All-Cause Mortality Attributable to Not Living with a Partner/Spouse

In this study, we also assessed the PAF for CVD and all-cause mortality associated with not living with a partner/spouse to illustrate the health benefits of living with a partner. The PAFs for CVD and all-cause mortality attributable to not living with a partner/spouse were comparable to the PAFs associated with living completely alone, suggesting that cohabiting with a partner or spouse can offer greater risk reduction. This highlights the role of partnership, likely attributable to the emotional support and companionship it provides, which can encourage healthier lifestyle choices and help mitigate stress. As no studies were found examining the associations (via odds ratio, relative risk, or hazard ratio) and PAFs for not living with a partner/spouse in relation to CVD and all-cause mortality, a direct comparison with our findings was not possible. Nonetheless, the insights derived from comparing the PAFs associated with living completely alone, as indicated in the previous section, can similarly be applied in this context.

### Public Health Implications

The study was based on a well-characterised prospective longitudinal study with adjudicated outcomes, CVD outcomes, and all-cause mortality. Our findings suggest significant implications for ageing policies, particularly given the marked gender differences in cardiovascular and mortality outcomes associated with living arrangements. In Australia and internationally, there is increasing utilisation of findings that advocate for the effective implementation of the Ageing in Place policy strategies.^52^ This policy aims to empower older adults to live independently in their own homes and avoid utilising more expensive hospitals and aged-care facilities. This policy focus is particularly relevant in the context of our findings, as the increased cardiovascular risks observed among women living alone may inadvertently lead to higher rates of hospital admission, a setting where secondary risks, such as hospital-acquired infections, become a concern.^53,54^ Ageing in place strategies use a combination of community resources to maintain quality of life, including family support, home-based services, and modifications to make housing more accessible and appropriate, in order to address complex issues that often emerge in later life including chronic health conditions, frailty, and changes in cognition. Our study reveals a critical challenge for these ageing in place policies: women, who are substantially more likely to live alone in later life, face significantly higher health risks from this living arrangement compared to men. The PAF indicates that 13.5% of cardiovascular events and 9.8% of mortality among women could be attributed to living alone, compared to just 2.9% and 3.6% respectively for men. This marked gender disparity suggests the need for a dual approach to policy and public health campaigns. First, for those choosing to live independently, enhanced support systems must specifically target women’s cardiovascular health risks. This requires public health campaigns that raise awareness among both health care providers and older adults about the gender-specific risks of living alone, leading to enhanced screening protocols and early intervention strategies. These campaigns should focus on practical steps for maintaining social connection and accessing community support, rather than simply highlighting risks.

Practical interventions should emphasise mechanisms that enhance social connection and community access, such as subsidised transportation, community link workers, and structured local activity programs. These could be further supported through technology-enabled social programs and integrated health monitoring, ensuring older women can effectively access community resources while maintaining independence. Second, ageing in place policies should expand to include opportunities for alternative living arrangements that maintain independence while providing built-in social support. International evidence suggests several viable models, including intergenerational housing programs,^55,56^ naturally occurring retirement communities,^57^ and co-housing models. Implementation requires coordinated action across health care, housing, and social services sectors, supported by public education campaigns that destigmatise shared living arrangements and highlight their potential health benefits. Pilot programs and partnerships with private sector stakeholders could test and refine these models in diverse Australian contexts.

Finally, increased funding for research focused on gender-specific impacts of social determinants is essential for developing evidence-based guidelines that cater to the distinct needs of both genders, ultimately improving health outcomes across populations. Priority areas include evaluating the effectiveness of various housing models in reducing cardiovascular risk, understanding how socioeconomic factors influence outcomes for women living alone, and examining long-term health trajectories. Establishing clear metrics for intervention success, particularly around cardiovascular events and mortality rates, will be crucial for guiding resource allocation and policy development.

### Limitations

Our study should be interpreted in the context of some potential limitations. First, the study population comprised healthy older adults living in the community, in which 99% of them self-identified as white. Thus, the generalisability of our findings to populations with different socioeconomic statuses and various ethnic groups might be limited and therefore, validation across diverse populations is necessary. Second, living alone collected at baseline was considered as the exposure, and this does not capture change over time. Future studies are warranted to consider living arrangements collected at multiple timepoints. Third, lacking data regarding duration of living alone and the quality and length of marriage/partnership is also a limitation. Finally, PAF is essential for quantifying how much a specific risk factor contributes to the overall burden of disease; public health officials and policymakers need to approach it with caution. The PAF is valuable for planning prevention strategies and allocating resources; however, its underlying assumption that eliminating a risk factor would completely prevent the related disease burden may be overly simplistic in real-world situations. The complex interplay of multiple risk factors and the possibility of compensatory behaviours mean that the true impact of removing a single risk factor may not align perfectly with PAF estimates.

## CONCLUSION

Living arrangements for older adults may have significant gender-specific impacts on CVD risk and all-cause mortality. Our study found statistically significant increased PAFs of CVD and all-cause mortality among older women who were living completely alone and not living with a partner/spouse. Future public health interventions should incorporate gender-specific interventions to effectively mitigate the heightened risk associated with living alone among older women.

## Supplementary Information

Below is the link to the electronic supplementary material.

Supplementary file1 (DOCX 55.0 KB)

## Data Availability

All relevant data are included in the manuscript or provided in the supplementary file. The ASPREE and ALSOP datasets are not publicly available due to the ongoing nature of the studies. However, access can be granted to partnering and external researchers with projects of appropriate scientific merit. Expressions of interest are coordinated through the ASPREE Access Management Site (AMS): https://aspree.org/aus/researchers/.
