# Supplementary material for: Gender-Specific Population Attributable Fractions for Cardiovascular Disease and All-Cause Mortality Associated with Living Arrangement in Community-Dwelling Older People
Source: J Gen Intern Med. 2025 Jun 25;40(11):2613–23. doi: 10.1007/s11606-025-09648-7 (PMC12405118; doi:10.1007/s11606-025-09648-7)
Supplement: Supplementary file 1 — Supplementary file1 (DOCX 55.0 KB) [file 11606_2025_9648_MOESM1_ESM.docx]

**Gender-Specific Population Attributable Fractions for Cardiovascular Disease and All-Cause Mortality Associated with Living Arrangement in Community-Dwelling Older People**

**Supplementary material**

**List of tables**

**Table S1.** Social determinants of health variables and the traditional risk factors used as covariates and their measurement

**Table S2.** Population attributable fractions (PAFs) for cardiovascular disease and all-cause mortality associated with living alone, calculated manually using Levin's formula.

**Table S1.** The traditional risk factors and social determinants of health variables used as covariates and their measurement

| Variables | Categorisation | Remark |
| --- | --- | --- |
| 1. Traditional risk factors | | |
| Gender | Men and women |  |
| Age | Age in years is measured as a continuous variable. |  |
| Hypertension | Hypertension was defined as treatment for high blood pressure or a blood pressure of more than 140/90 mmHg at trial entry [1]. |  |
| Diabetes | The presence of diabetes was based on participants' reports of diabetes mellitus or a fasting glucose level of at least 126 mg/dL (≥7 mmol/L) or receipt of treatment for diabetes [1]. |  |
| Dyslipidaemia | Dyslipidemia was defined as the receipt of cholesterol-lowering medication or as a serum cholesterol level of at least 212 mg/dL (≥5.5 mmol/L) or as a low-density lipoprotein level of more than 160 mg/dL (>4.1 mmol/L) [1]. |  |
| Urine albumin-to-creatinine ratio (ACR) | The urine albumin to creatinine ratio, expressed in mg/mmol, is utilised as a continuous variable. |  |
| Estimated glomerular filtration rate (eGFR) | Estimated glomerular filtration rate in ml/min/1.73m^2^ as a continuous variable |  |
| Current smoking | Whether the participants were smokers or not during enrollment. |  |
| 1. Social Determinants of Health | | |
| 1. *Economic Stability* | | |
| Current employment status | Employed (Full/Part-time) and not employed |  |
| Household income (per year) | High income (≥$50,000, those preferred not to answer considered here) and Low (<$50,000) |  |
| Homeownership | Defined as owned if either the participant or spouse/partner has a house and not otherwise |  |
| 1. *Education Access and Quality* | | |
| Education status | High (>12 years of education) and Low (≤12 years of education) |  |
| First language | English vs. Not English |  |
| 1. *Social and Community Context* | | |
| Social network | Based on the Lubben Social Network Scale-6 (LSNS-6). LSNS-6 consists of six questions, each scored from 0 to 5, concerning the number of relatives and friends (separately) a person contacts at least monthly [2].  The questions are:  1) How many relatives and friends do you see or hear from at least once a month?  2) How many relatives and friends do you feel comfortable discussing private matters with?  3) How many relatives and friends do you feel close enough to that you could ask them for help?  The total score is the sum of the six items, with five possible responses each, ranging from 0 to 30.  An optimal cutoff point to differentiate high social engagement from low social engagement (social isolation) was determined using an X-tile program, supported by a diagnostic plot. This cutoff point aligns with the one proposed by Lubben et al., where a score of 12 or lower indicates social isolation. Thus, our cutoff point for considering someone socially isolated (low social network) is 12 or lower for men and 13 or lower for women. | Original responses:   1. None 2. One 3. Two 4. Three or four 5. Five through eight 6. Nine or more |
| Social participation | Consistent with other research [3, 4], social participation was gauged using five specific items or questions.  How frequently do you:   1. Go to a club, local organisation, neighbourhood or other small group? 2. Go to church, temple or other places of worship, or take part in related activities? 3. Do you go to museums, galleries or exhibitions? 4. Do you go to an education class? 5. Do you go to the cinema, theatre or other social/sporting entertainment?   In this study, social participation was categorised as adequate/high if individuals belonged to or participated in any of the activities at least once per month, and as inadequate/low if they did not. | Original Responses:   1. Never 2. Less than once a month 3. 1-3 times a month 4. Once a week or more 5. Most days |
| Volunteering | Four variables were utilised to assess volunteering: providing care for other adults in the past year, offering primary care for sick children during adulthood, volunteering for babysitting in the past year, and engaging in unpaid volunteer work (excluding babysitting and caregiving). Volunteering was then classified as "Yes" if the participant engaged in any of the activities, and "No" if they did not. | Original responses: Volunteering primary care for sick children and currently doing unpaid volunteer work had binary responses (yes-no type). Babysitting and care provider for adults during the past year was measured using 0-3 Likert Scales.   1. Never 2. Occasionally (once a week) 3. Often (more than once a week) 4. Every/most days   These two variables were recoded into yes (participated at least once a week) and no (never participated). |
| Hobby engagement | A composite variable was assessed using nine activities: listening to the radio or music, reading books, newspapers, or magazines, playing games, engaging in puzzles or crosswords, crafting, writing letters, using a computer, cooking, and painting or drawing. Initially, each activity was recoded from five possible responses to a binary scale: at least once a month (Never and Rarely; coded as "0") and less than once a month (coded as "1"). Subsequently, the composite score (ranges from 0-9) was recoded as ≤3 (Less engaged) and >3 (highly engaged). |  |
| Expectations and attitudes (optimism–pessimism) | Measured based on the six items of the Life Orientation Test-Revised (LOT-R) [5]:   1. In uncertain times, I usually expect the best ®. 2. If something can go wrong for me, it will. 3. I’m always optimistic about my future ®. 4. I hardly ever expect things to go my way. 5. I rarely count on good things happening to me. 6. Overall, I expect more good things to happen to me than bad ®.   Each item is rated on a 5-point scale. A composite score is then created, ranging from 0-24, with higher scores indicating greater levels of optimism. Finally, it was binary coded as ≤13 (less optimistic) and >13 (highly optimistic) | Original responses:   1. Strongly agree. 2. Agree a little 3. Neither agree nor disagree 4. Disagree a little 5. Strongly disagree   ® Reverse coded |
| Difficulties and stressful life events | Ten stressful life events from the past year were assessed with 'yes' or 'no' responses. These included: 1) death of a spouse/partner, 2) serious illness in a spouse/partner, 3) death or serious illness in family members or close friends, 4) major financial problems, 5) divorce or breakup with a spouse or partner, 6) divorce or breakup in family members or close friends, 7) major conflict with children or grandchildren, 8) major accidents, disasters, muggings, unwanted sexual experiences, robberies, or similar events, 9) job loss or retirement in family members or close friends, and 10) death of a pet. Following previous literature [6], we computed a continuous stressful life events variable by summing the number of events and categorised the score accordingly into three categories (number of past-year adverse events: 0 events, 1 event, 2 events, and 3 or more events). |  |
| Depressive symptoms | The Center for Epidemiologic Studies Depression Scale (CESD-10) was utilised in this study [7]. Then a score of 8 or higher was used to indicate the presence of depression. |  |
| 1. *Neighbourhood and built environment* | | |
| Remoteness | Participants' rurality is categorised into three areas: Major cities, Inner regions, and Outer regions of Australia. |  |
| Socio-Economic Indexes for Areas (SEIFA) | The Socio-Economic Indexes for Areas-Index of Relative Socioeconomic Advantage and Disadvantage (SEIFA-IRSAD) was determined using the residential postcodes of participants, drawing on data from the 2011 Australian Census. The calculation of the SEIFA-IRSAD index takes into account factors such as annual household income, education, occupation, internet access, and the rental rates of dwellings in a given area. The study took into account the SEIFA-IRSAD scores, which were calculated in quintiles. |  |
| Transport (satisfaction) | Transport satisfaction was assessed with a single item on a 5-point scale. In this study, responses were recoded as "Satisfied" if participants were very satisfied or moderately satisfied, and as "Not Satisfied" if they were very dissatisfied, somewhat dissatisfied, or neither. | Original responses:   1. Very satisfied 2. Moderately satisfied 3. Neither satisfied nor dissatisfied 4. Somewhat dissatisfied 5. Very dissatisfied |
| 1. *Healthcare quality and access* | | |
| Health insurance | Health care insurance is categorised as a binary variable, coded as either insured (includes concession, department of veterans’ affairs (DVA), and private insurance) or not insured. |  |

**Table S2.** Population attributable fractions (PAFs) for cardiovascular disease and all-cause mortality associated with living alone, calculated manually using Levin’s formula.

| Study^a^, year | Gender | Measures of association (MoA) | Prevalence of living alone | Follow-up years | PAF based on Levin’s formula [8]^b^ | Outcome | Age and population |
| --- | --- | --- | --- | --- | --- | --- | --- |
| Kitamura et al., 2013 [9] | Men | HR = 1.34 | 0.16 | 2 | 0.051 | Major adverse cardiovascular event (MACE) and total deaths | Median age: living alone = 69 years; and not living alone = 66 years, among patients with acute myocardial infarction. |
|  | Women | HR = 1.31 | 0.25 |  | 0.072 |  |  |
| Redfors et al., 2016 [10] | Men | HR = 3.47 | 0.28 | 8.8 | 0.410 | All-cause mortality | Age less than 70 years, among patients with ischemic stroke. |
|  | Women | HR = 1.28 | 0.32 |  | 0.082 |  |  |
| Schmaltz et al., 2007 [11] | Men | HR = 2 | 0.13 | 3 | 0.115 | All-cause mortality | Mean age = 65.4 years, among patients with acute myocardial infarction. |
|  | Women | HR = 1.2 | 0.31 |  | 0.058 |  |  |
| Avlund et al., 1998 [12] | Men | OR = 2.11 | 0.04 | 11 | 0.043 | All-cause mortality | 70-year-old, community-dwelling. |
|  | Women | OR = 1.43 | 0.12 |  | 0.049 |  |  |
| Kandler et al., 2007 [13] | Men | HR = 1.96 | 0.07 | 11.4 | 0.063 | All-cause mortality | Aged 45-74 years, community-dwelling. |
|  | Women | HR = 1.11 | 0.18 |  | 0.019 |  |  |
| Kilpi et al., 2015 [14] | Men | HR = 1.18 | 0.14 | 12 | 0.025 | Incident myocardial infarction | Aged 40-60 years, community-dwelling. |
|  | Women | HR = 1.16 | 0.14 |  | 0.022 |  |  |
| NG et al., 2020 [15] | Men | HR = 1.38 | 0.22 | 26 | 0.077 | All-cause mortality | Aged 50-60 years, community-dwelling. |
|  | Women | HR = 1.27 | 0.21 |  | 0.054 |  |  |
| Pimouguet et al., 2015 [16] | Men | HR = 1.44 | 0.39 | 6 | 0.146 | All-cause mortality | Participants aged ≥66 years, community-dwelling. |
|  | Women | HR = 1.19 | 0.72 |  | 0.120 |  |  |
| Scafato E et al., 2008 [17] | Men | HR = 1.42 | 0.08 | 10 | 0.034 | All-cause mortality | Aged 65–84 years, both community-dwelling and institutionalised. |
|  | Women | HR = 1.05 | 0.27 |  | 0.013 |  |  |
|  | Women | HR = 0.73 | 0.49 |  | - |  |  |
| NG et al., 2015 [18] | Men | HR = 2.36 | 0.22 | 8 | 0.230 | All-cause mortality | Older adults aged 55 years and above, the general population. |
|  | Women | HR = 1.14 | 0.78 |  | 0.098 |  |  |
|  | Women | HR = 0.42 | 0.07 |  | - |  |  |
| Lund et al., 2000 [19] | Men | OR = 1.04 | 0.30 | 4 | 0.012 | All-cause mortality | Aged 70-95 years, among the general population. |
|  | Women | OR = 2.57 | 0.68 |  | 0.516 |  |  |
| Dahl et al., 2021[20] | Men | HR = 1.37 | 0.37 | 12.8 | 0.120 | All-cause mortality | Aged 50 to 79 years, among patients with hip fracture |
|  | Women | HR = 1.23 | 0.43 |  | 0.090 |  |  |

^a^ II did the literature search and extracted the data.

^b^ The population attributable fraction (PAF) is a key metric for assessing the disease burden linked to a specific risk factor. It can be calculated using either Levin's or Miettinen's formula [21]. In our case, we employed Levin's formula ((Prevalence of exposure*(MoA-1))/(Prevalence of exposure*(MoA-1)+1)) to estimate the PAF for cardiovascular disease and all-cause mortality associated with living alone. This approach was adopted to facilitate a rough comparison of our findings with findings from existing studies. However, it should be noted that Levin's formula has inherent limitations, and as such, the calculated PAF may not fully reflect the true value.

**Abbreviations:** HR, hazard ratio; OR, odds ratio; PAF, population attributable fraction; RR, relative risk

**References**

1. McNeil JJ, Wolfe R, Woods RL, Tonkin AM, Donnan GA, Nelson MR, et al. Effect of Aspirin on Cardiovascular Events and Bleeding in the Healthy Elderly. N Engl J Med. 2018;379(16):1509-18.

2. Lubben J, Blozik E, Gillmann G, Iliffe S, von Renteln Kruse W, Beck JC, et al. Performance of an Abbreviated Version of the Lubben Social Network Scale Among Three European Community-Dwelling Older Adult Populations. The Gerontologist. 2006;46(4):503-13.

3. Hwang J, Park S, Kim S. Effects of Participation in Social Activities on Cognitive Function Among Middle-Aged and Older Adults in Korea. Int J Environ Res Public Health. 2018;15(10).

4. Floud S, Balkwill A, Canoy D, Reeves GK, Green J, Beral V, et al. Social participation and coronary heart disease risk in a large prospective study of UK women. Eur J Prev Cardiol. 2016;23(9):995-1002.

5. Glaesmer H, Rief W, Martin A, Mewes R, Brähler E, Zenger M, et al. Psychometric properties and population‐based norms of the Life Orientation Test Revised (LOT‐R). British journal of health psychology. 2012;17(2):432-45.

6. Berntson J, Patel JS, Stewart JC. Number of recent stressful life events and incident cardiovascular disease: Moderation by lifetime depressive disorder. Journal of Psychosomatic Research. 2017;99:149-54.

7. Andresen EM, Malmgren JA, Carter WB, Patrick DL. Screening for depression in well older adults: Evaluation of a short form of the CES-D. American journal of preventive medicine. 1994;10(2):77-84.

8. Levin ML. The occurrence of lung cancer in man. Acta Unio Int Contra Cancrum. 1953;9(3):531-41.

9. Kitamura T, Sakata Y, Nakatani D, Suna S, Usami M, Matsumoto S, et al. Living alone and risk of cardiovascular events following discharge after acute myocardial infarction in Japan. Journal of Cardiology. 2013;62(4):257-62.

10. Redfors P, Isaksén D, Lappas G, Blomstrand C, Rosengren A, Jood K, et al. Living alone predicts mortality in patients with ischemic stroke before 70 years of age: a long-term prospective follow-up study. BMC Neurology. 2016;16(1):80.

11. Schmaltz HN, Southern D, Ghali WA, Jelinski SE, Parsons GA, King KM, et al. Living Alone, Patient Sex and Mortality After Acute Myocardial Infarction. Journal of General Internal Medicine. 2007;22(5):572-8.

12. Avlund K, Damsgaard MT, Holstein BE. Social relations and mortality. An eleven year follow-up study of 70-year-old men and women in Denmark. Social Science & Medicine. 1998;47(5):635-43.

13. Kandler U, Meisinger C, Baumert J, Löwel H, the KSG. Living alone is a risk factor for mortality in men but not women from the general population: a prospective cohort study. BMC Public Health. 2007;7(1):335.

14. Kilpi F, Konttinen H, Silventoinen K, Martikainen P. Living arrangements as determinants of myocardial infarction incidence and survival: A prospective register study of over 300,000 Finnish men and women. Soc Sci Med. 2015;133:93-100.

15. Ng N, Santosa A, Weinehall L, Malmberg G. Living alone and mortality among older people in Västerbotten County in Sweden: a survey and register-based longitudinal study. BMC Geriatrics. 2020;20(1):7.

16. Pimouguet C, Rizzuto D, Schön P, Shakersain B, Angleman S, Lagergren M, et al. Impact of living alone on institutionalization and mortality: a population-based longitudinal study. European Journal of Public Health. 2015;26(1):182-7.

17. Scafato E, Galluzzo L, Gandin C, Ghirini S, Baldereschi M, Capurso A, et al. Marital and cohabitation status as predictors of mortality: A 10-year follow-up of an Italian elderly cohort. Social Science & Medicine. 2008;67(9):1456-64.

18. Ng TP, Jin A, Feng L, Nyunt MSZ, Chow KY, Feng L, et al. Mortality of older persons living alone: Singapore Longitudinal Ageing Studies. BMC Geriatrics. 2015;15(1):126.

19. Lund R, Modvig J, Due P, Evald Holstein B. Stability and change in structural social relations as predictor of mortality among elderly women and men. European Journal of Epidemiology. 2000;16(12):1087-97.

20. Dahl C, Holvik K, Meyer HE, Stigum H, Solbakken SM, Schei B, et al. Increased Mortality in Hip Fracture Patients Living Alone: A NOREPOS Study. Journal of Bone and Mineral Research. 2021;36(3):480-8.

21. Lin C-K, Chen S-T. Estimation and application of population attributable fraction in ecological studies. Environmental Health. 2019;18(1):52.
